# Educational interventions for health professionals managing chronic obstructive pulmonary disease in primary care

**DOI:** 10.1017/S1463423623000609

**Published:** 2024-02-12

**Authors:** Daksha Trivedi

**Affiliations:** Centre for Research in Public Health and Community Care, University of Hertfordshire, Hatfield, UK

**Keywords:** chronic obstructive pulmonary disease, COPD, education, health professionals, interventions, primary care, programmes

## Review question

Primary: To examine evaluations of educational interventions for health professionals who manage COPD in primary care.

## Relevance to primary care and nursing

Primary health professionals including specialist-trained nurses have a pivotal role in supporting people with chronic obstructive pulmonary disease (COPD) (National Institute for Health and Care Excellence, 2018). This includes treatment and support, vaccinations, pulmonary rehabilitation, and co-developing personalised self-management plans. Educational interventions offered to health professionals as part of their continuing professional development (e.g. courses and workshops and practice-based activities in various formats) have the potential to improve the knowledge, skills, attitudes and behaviour of health professionals, and patient-related outcomes.

## Characteristics of the evidence

This Cochrane review contains 22 cluster randomised controlled trials (RCT) and 16 RCTs conducted in primary care involving 4,936 health professionals and 71,085 patient participants (Cross *et al.*, 2022). They were conducted in the United States (*n* = 6), Canada (*n* = 1), Australia (*n* = 6), Europe (*n* = 17), Asia (*n* = 4), South Africa (*n* = 3) and Brazil (*n* = 1). Key elements of interventions delivered to health professionals included education, toolkits, guidelines, spirometry training and feedback/mentorship, which aimed to improve their skills and knowledge in managing COPD. They were compared with no intervention or with disseminated printed management guideline.

## Summary of key evidence

Follow-up ranged from six months to two years. Pooled evidence ranged from very low certainty to moderate certainty judged using The Grading of Recommendations Assessment, Development and Evaluation, and most studies had heterogeneity, with unclear or high risk of bias across multiple domains.

Primary and secondary outcomes are indicated below. Number of studies and number of participants (N) are given in parentheses. They are presented as risk ratios (Relative risk, RR) for binary outcomes. Effect sizes from meta-analysis of pooled studies are indicated where available and presented as mean difference with 95% confidence interval (CI), and significance levels (*p* value) are shown where reported.

## Primary outcomes

### Proportion of COPD diagnoses confirmed with spirometry

Evidence from four low-quality studies (*n* = 1,896) gave mixed results. Of these, one educational workshop for general practitioners (GPs) and practice nurses reported 16/79 (20.3%) versus 1/408 (0.2%). Another structured disease management programme increased the number of patients having spirometry at least once a year (RR 1.36, 95% CI 1.09 to 1.70, *n* = 458 versus 1.07, 95% CI 0.85 to 1.34, *n* = 376), and the rest did not show clear impacts.

### Proportion of patients with COPD referred to, participating in or having completed pulmonary rehabilitation

Evidence from four studies (*n* = 625) of low quality gave mixed results. Blended face-to-face and online education to physicians/GPs increased referrals in two studies (14/117, 12% versus 4/125, 3%); and physician referrals (66.4%, *n* = 19 physicians versus 40.9%, *n* = 21 physicians). Team-based COPD education showed no significant impact at 12 months.

### Proportion of patients with COPD prescribed respiratory medications consistent with recommended guidelines

Evidence from 12 low-quality studies (*n* = 52,899, follow-up range 3 months to over 4 years) involving education for prescribers, guideline provision, central case management resources and/or increased prescribing provisions for nurse practitioners was mixed. Only six reported significant changes in lower prescribing indicating appropriate prescribing (*n* = 2 studies; 15/101 (14.9%) versus 29/67 (43.3%); and mean 3.63 (standard deviation (SD) 2.96), *n* = 72 versus 4.12 (SD 3.10, *n* = 51)).

- Inhaled corticosteroids (137/1,000, 13.7% versus 77/999, 7.7%, *P* = 0.006).
- Guidelines with feedback resulted in greater prescription of penicillins, but lower prescription of quinolones for respiratory tract infections compared to usual care.
- Training in COPD plus a comprehensive health management programme for patients; education plus use of COPD Care Manager online module with access to central case management resources resulted overall in an increased frequency of prescribing of various medications.

## Secondary outcomes

For secondary outcomes, there was a small positive impact on influenza vaccination rates with little impact against pneumococcal infection. There was overall little significant effect on health-related quality of life and frequency of COPD exacerbations. Only one intervention involving clinicians’ training and patients (*n* = 1,222, moderate-quality study) receiving three dimensions of health review by nurse, pharmacist and physician showed a higher proportion of patients being ‘very satisfied with care’ compared to usual care (345/614, 56% versus 236/608, 39%; *P* = 0.0014)

## Implications for practice

Low-quality evidence indicates uncertainty about the benefits or applications of educational interventions to clinical practice. It suggests a need to develop and evaluate interventions that adopt adult learning principles and are tailored to local COPD guidelines.

## Implications for research

Larger high-quality studies are needed to better understand the impact of educational interventions for health professionals. Research should employ theoretical frameworks for developing and implementing complex interventions. Developing interventions that focus on action, audit and feedback, reminders and educational outreach, with more consistent reporting of relevant outcomes, are needed.
